# Adenosine Triphosphate Release and P2 Receptor Signaling in Piezo1 Channel-Dependent Mechanoregulation

**DOI:** 10.3389/fphar.2019.01304

**Published:** 2019-11-06

**Authors:** Linyu Wei, Fatema Mousawi, Dongliang Li, Sébastien Roger, Jing Li, Xuebin Yang, Lin-Hua Jiang

**Affiliations:** ^1^Sino-UK Joint Laboratory of Brain Function and Injury and Department of Physiology and Neurobiology, Xinxiang Medical University, Xinxiang, China; ^2^School of Biomedical Sciences, Faculty of Biological Sciences, University of Leeds, Leeds, United Kingdom; ^3^Department of Physiology, Sanquan College of Xinxiang Medical University, Xinxiang, China; ^4^EA4245, Transplantation, Immunology and Inflammation, Faculty of Medicine, University of Tours, Tours, France; ^5^Lingnan Medical Research Centre, School of Medicine, Guangzhou University of Chinese Medicine, Guangzhou, China; ^6^Department of Oral Biology, Faculty of Medicine and Health, University of Leeds, Leeds, United Kingdom

**Keywords:** mechanical stimuli, mechanosensitive cells, Piezo1 channel, adenosine triphosphate release, P2 receptors

## Abstract

Organs and tissues and their constituent cells are physiologically submitted to diverse types of mechanical forces or stress, one common sequence of which is release of intracellular ATP into extracellular space. Extracellular ATP is a well-established autocrine or paracrine signaling molecule that regulates multiple cell functions and mediates cell-to-cell communications *via* activating the purinergic P2 receptors, more specifically, ligand-gated ion channel P2X receptors and some of the G-protein-coupled P2Y receptors. The molecular mechanisms that sense mechanical and transduce forces to trigger ATP release are poorly understood. The Piezo1, a newly identified mechanosensing ion channel, shows widespread expression and confers mechanosensitivity in many different types of cells. In this mini-review, we briefly introduce the Piezo1 channel and discuss the evidence that supports its important role in the mechanoregulation of diverse cell functions and, more specifically, critical engagement of ATP release and subsequent P2 receptor activation in Piezo1 channel-dependent mechanoregulation. Such ATP release-mediated coupling of the Piezo1 channel and P2 receptors may serve a signaling mechanism that is more common than we currently understand in transducing mechanical information to regulation of the attendant cell functions in various organs and tissues.

## Introduction

**Figure 1:**
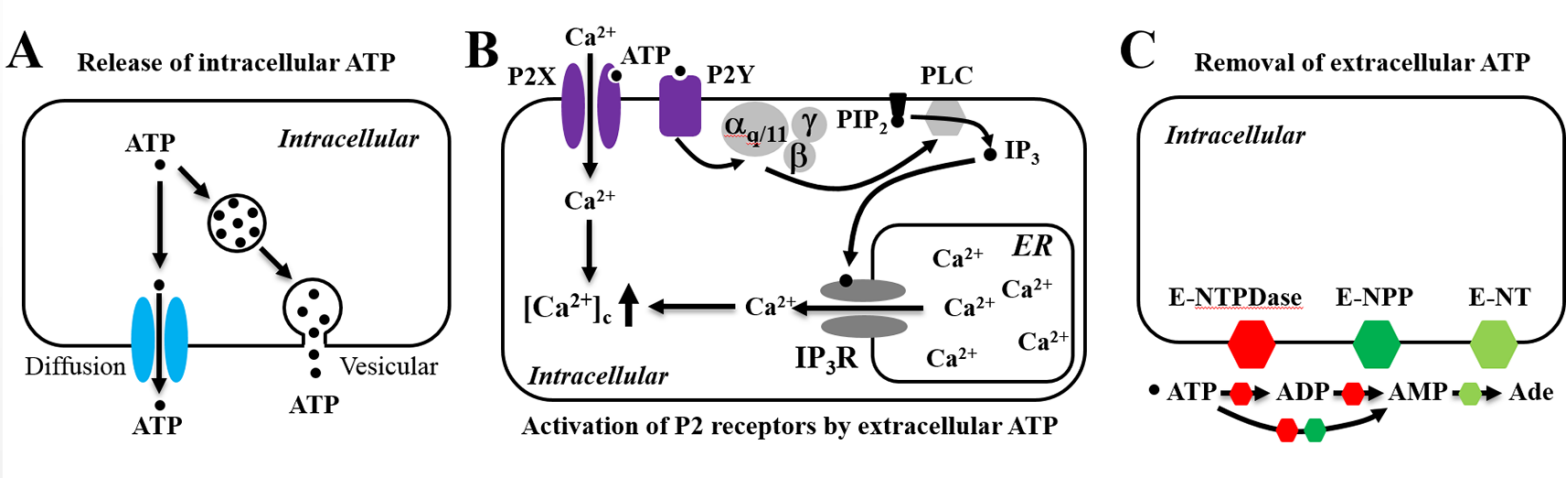
Schematic illustration of the adenosine triphosphate (ATP)-based signaling system in mammalian cells. The ATP-based signaling system comprises of the following three principal components. **(A)** Release of intracellular ATP, which occurs *via* exocytosis (vesicular) and/or diffusion through many different types of ion channels. **(B)** Extracellular ATP as an autocrine or paracrine signal activating ligand-gated ion channel P2X receptors and/or G-protein-coupled P2Y receptors. ATP gates all P2X receptor ion channels, allowing extracellular Ca^2+^ influx. Alternatively, ATP activates the P2Y receptors, mainly P2Y_1_, P2Y_2_, and P2Y_11_, leading to sequential activation of G_α,q/11_, phospholipase C (PLC), conversion of membrane lipid phosphatidylinositol 4,5-bisphosphate (PIP_2_) to inositol triphosphate (IP_3_) and diacylglycerol (not depicted), activation of the IP_3_ receptor (IP_3_R), and Ca^2+^ release from the endoplasmic reticulum (ER). **(C)** Termination of the actions of ATP by converting to ADP, AMP, and adenosine (Ade) by ecto-nucleotidases, including ecto-nucleoside triphosphate diphosphohydrolase (E-NTPDase), ecto-nucleotide pyrophosphatase/phosphodiesterase (E-NPP), and by ecto-5’-nucelotidase (E-NT).

Adenosine triphosphate (ATP), while it is best known for its intracellular role as the cellular energy source, gains increasing recognition as an extracellular signaling molecule when it is released into extracellular spaces. In mammalian cells, the ATP-based signaling system comprises of three principal components: release of intracellular ATP into the extracellular space, activation of the ligand-gated ion channel P2X receptors and/or G-protein-coupled P2Y receptors for extracellular ATP, and removal of extracellular ATP to terminate its action by a broad family of ATP-scavenging ecto-nucleotidases that convert ATP to ADP, adenosine monophosphate, or adenosine (**Figure 1**) (Verkhratsky and Burnstock, 2014; Jiang et al., 2017a). This system represents one of the most common signaling mechanisms regulating cell functions and mediating cell-to-cell communications and plays a critical role in a wide range of physiological processes, such as hearing, tasting, nociception, immune responses, muscle contraction, learning, and memory. There exists a large volume of evidence that alterations in such an ATP-based signaling system contribute in the pathogenesis and progression of diverse conditions, ranging from hearing loss, pain, inflammatory diseases, hypertension, neurodegenerative diseases, and psychotic disorders to cancer metastasis (North, 2002; Fields and Burnstock, 2006; Khakh and North, 2006; Abbracchio et al., 2009; Surprenant and North, 2009; Zimmermann et al., 2012; Caseley et al., 2014; Roger et al., 2015; Cekic and Linden, 2016; Krugel, 2016; Alves et al., 2018; Di Virgilio et al., 2018; Wei et al., 2018).

It is conceivable that ATP easily leaks from damaged or dying cells as a danger signal alerting tissue damage and inflammation. However, decades of studies provide clear evidence to show that many types of cells can release ATP without compromise in cell viability and a variety of physical and chemical signals or stimuli can induce non-lytic release of ATP. Two general release pathways, namely, vesicular and diffusion, have been proposed for efflux of intracellular ATP (Verkhratsky and Burnstock, 2014). However, the molecular mechanisms mediating ATP release are still not fully elucidated, in part due to that such mechanisms appear to be diverse and cell-type specific. Furthermore, many types of cells are equipped with multiple ATP release mechanisms and deploy them according to the nature of the incoming stimuli. Vesicular release *via* exocytosis represents the major mechanism by which neurons release ATP into the synaptic cleft in the peripheral and central nervous systems (Pankratov et al., 2006; Abbracchio et al., 2009; Masuda et al., 2016). Vesicular ATP release *via* exocytosis has been also described in astrocytes (Chen et al., 2013; Lalo et al., 2014), urothelial cells (Nakagomi et al., 2016), neutrophils (Harada et al., 2018), and pancreatic β-cells (Geisler et al., 2013; Sakamoto et al., 2014). In this regard, it is worth mentioning that the vesicular nucleotide transporter (VNUT) plays a critical role in mediating vesicular storage and thereby subsequent release of ATP (Sawada et al., 2008) (for more details, see Moriyama et al., 2017; Miras-Portugal et al., 2019). On the other hand, several distinctive types of ion channels have been suggested to act as conduits permitting diffusion of ATP out of cells. The volume-regulated anion channel (VRAC) has been identified to mediate non-synaptic release of ATP from axons in response to action potential-induced swelling (Fields and Ni, 2010). The pannexin hemi-channels, calcium homeostasis modulator 1 (CALHM1), cystic fibrosis transmembrane conductance regulator (CFTR), maxi-anion channel, and P2X7 receptor as well as the VRAC have been reported to mediate or regulate ATP efflux from a variety of non-neuronal cells. For detailed discussion of these ATP release mechanisms, the readers can consult recently published reviews (e.g., Verkhratsky and Burnstock, 2014; Taruno, 2018).

Cells are physiologically submitted to diverse types of mechanical forces or stress and virtually all types of cells exhibit a mechanosensitivity. They can sense external or “outside-in” mechanical forces, for example, fluid flow-induced shear stress, osmotic stress, and pressure-induced membrane stretch (Nourse and Pathak, 2017). Cells can also generate traction forces *via* actin-myosin interactions at the focal adhesion zones and apply such “inside-out” mechanical forces to survey the mechanical and geographical properties of extracellular matrix and cell-supporting substrates (Nourse and Pathak, 2017; Ellefsen et al., 2019). Importantly, cells are able to convert mechanical forces into intracellular signals and even integrate mechanical information into the genomic blueprint (Choi et al., 2019), indicating that mechanical stimulation can have long-term effects as well as short-term effects on cell functions. Mechanical stimuli are long known as a potent trigger for non-cytolytic release of ATP both *in vivo* and *in vitro*, and accumulating evidence supports that ATP release and subsequent activation of the P2 receptors act as a crucial signal transduction mechanism in the mechanoregulation of cell functions (Petruzzi et al., 1994; Riddle et al., 2007; Wan et al., 2008; Yu et al., 2010; Sun et al., 2013; Miyamoto et al., 2014; Weihs et al., 2014; Cinar et al., 2015; Wang et al., 2016; Iring et al., 2019). However, the molecular identity of the mechanosensor that directly detects the mechanical forces and triggers ATP release remained elusive. The Piezo1 ion channel has emerged as an intrinsically mechanically activated Ca^2+^-permeable cation channel that confers cells with an ability of sensing diverse forms of mechanical stimuli (Murthy et al., 2017; Wu et al., 2017; Xiao, 2019). Furthermore, a large number of recent studies have shown an important role of the Piezo1 channel in the mechanoregulation of a wide range of physiological and pathological functions (Murthy et al., 2017; Wu et al., 2017; Xiao, 2019). Of interest, accumulating evidence supports that ATP release and P2 receptor signaling are important in mediating Piezo1 channel-dependent mechanoregulation. The two separate domains of investigation thus need to join forces in order to develop a full and mechanistic understanding of mechanoregulation. The aim of this mini-review is to introduce the Piezo1 channel and discuss the recent studies that provide evidence to support its crucial role in several types of mechanosensitive cells in the induction of ATP release and subsequent activation of the P2X or P2Y receptors and the mechanoregulation of the attendant cell functions. With increasing evidence to show their overlapping expression in many different types of mechanosensitive cells, the Piezo1 channel and P2 receptors, *via* coupling by ATP, may serve as a signaling mechanism that is more common than we currently understand in transducing the mechanical information to functional regulation.

### A Brief Introduction to the Piezo1 Channel

The Piezo1 protein (also known as Fam38a) was identified to form the mechanically activated ion channel mediating pressure-induced ionic currents in mouse neuroblastoma Neuro2A cells (Coste et al., 2010; Coste et al., 2012; Syeda et al., 2016). In the same seminal study, a homologue protein, Piezo2 (also known as Fam38b), was found to express in a subset of mouse dorsal root ganglia neurons and can also form a mechanically activated ion channel with comparatively faster inactivation kinetics. The Piezo proteins are large in size, being ∼2,500–2,800 amino acid residues long and with predicted molecular weights of ∼290-320 kDa for the mouse and human proteins. They are predicted to have a unique membrane topology composed of 38 transmembrane segments and intracellular N- and C-termini (Zhao et al., 2019). Several structures containing the core parts of the mouse Piezo1 channel have been recently determined by cryo-electron microscopy (Saotome et al., 2018; Zhao et al., 2018; Wang et al., 2019). These structures reveal a trimeric assembly and a three-bladed propeller-like architecture of the Piezo1 channel. For further structural details, the readers can consult recently published reviews (e.g., Murthy et al., 2017; Xiao, 2019; Zhao et al., 2019).

Studies have demonstrated wide expression of the Piezo1 channel that enables many different types of cells to sense a diversity of “outside-in” mechanical forces, including indentation, membrane stretch, shear stress, osmotic stress, ultrasound, and compression (Coste et al., 2010; Li et al., 2014; Miyamoto et al., 2014; Pathak et al., 2014; Ranade et al., 2014; Jin et al., 2015; Lewis and Grandl, 2015; Syeda et al., 2016; Wang et al., 2016; Gao et al., 2017; Wu et al., 2017). There is also compelling evidence to suggest that the Piezo1 channel can be activated by traction forces (Pathak et al., 2014; Murthy et al., 2017; Nourse and Pathak, 2017; Ellefsen et al., 2019). Thus, two different, so-called “force-from-lipids” and “force-from-filaments,” mechanisms have been proposed for mechanical activation of the Piezo1 channel (Murthy et al., 2017). In the “force-from-lipids” mechanism, mechanical forces introduce membrane tension that leads to reorganization of lipids within and surrounding the channel proteins. The resultant alterations in the membrane lipid-channel protein interactions induce the channel to open. This gating mechanism has gained support from a recent study (Lin et al., 2019). The “force-from-filaments” mechanism proposes that the interactions between the channel and extracellular matrix or intracellular cytoskeletal proteins provoke conformational changes leading to the channel opening.

The mechanically activated ion channels are less amenable to electrophysiological studies as compared to the ion channels activated by other modalities, such as changes in membrane potential, temperature, or chemical ligands. This is in part because of the unease of applying mechanical stimuli to cells under the experimental settings and the challenge of accurately determining the mechanical forces inducing the channel activation (Parpaite and Coste, 2017). Yoda1, a synthetic chemical, selectively activates the Piezo1 channel with an EC_50_ (the concentration evoking 50% of the maximal response) of 2.5–27 µM, determined by measuring Yoda1-induced Ca^2+^ responses in cells expressing the recombinant mouse and human Piezo1 channels (Syeda et al., 2015; Evans et al., 2018). The discovery of Yoda1 has made it technically more approachable to the study of the Piezo1 channel under *in vitro* conditions. Grammostola spatulata mechanotoxin 4 (GsMTx4), a 34-amino acid peptide isolated from the venom of a tarantula spider and known to block mechanically activated currents (Suchyna et al., 2000), has been shown to inhibit the Piezo1 channel in the low micromolar concentrations (Bae et al., 2011; Bagriantsev et al., 2014). Mechanistically, GsMTx4 acts on the extracellular side as a channel gating modifier to modulate the arrangements of membrane lipids in the surroundings of the channel protein and thereby decreases the efficiency of force transduction from the lipid bilayer to the channel (Suchyna et al., 2000; Gnanasambandam et al., 2017). Ruthenium red (RR), a polycationic ion, can also inhibit the Piezo1 channel-mediated mechanically activated currents with an IC_50_ (the concentration causing 50% inhibition of the response) of 5.4 µM, which was shown at the *Drosophila* Piezo channel, and RR is thought to be an open channel blocker (Coste et al., 2012). Gadolinium ion (Gd^3+^) in the micromolar concentrations is known to inhibit the Piezo1 channel (Cinar et al., 2015). These negative allosteric modulators or inhibitors are lack of the specificity towards the Piezo1 channel (Bowman et al., 2007). Nonetheless, they provide useful pharmacological tools, in combination with genetic means, to better understand the role of the Piezo1 channel in physiological and pathological processes.

The expression of the Piezo1 channel has been shown in an increasing number of cell types in various tissues and organs, including neurons, astrocytes, smooth muscle cells, endothelial cells, epithelial cells, red blood cells, immune cells, periodontal ligament cells, neural progenitor cells, mesenchymal stem cells, and embryonic stem cells (e.g., Li et al., 2014; Pathak et al., 2014; Jin et al., 2015; Gudipaty et al., 2017; Murthy et al., 2017; Del Marmol et al., 2018; Friedrich et al., 2019; Mousawi et al., 2019; Solis et al., 2019; Song et al., 2019; Velasco-Estevez et al., 2019). The Piezo1 channel is mainly located in the plasma membrane (Coste et al., 2010; Coste et al., 2012; Miyamoto et al., 2014; Hung et al., 2016). Some evidence suggests that the Piezo1 channel is also present in the membrane of endoplasmic reticulum (McHugh et al., 2010) and in the cytoplasmic compartments near the nucleus (Miyamoto et al., 2014) and nuclear envelope (Gudipaty et al., 2017). A large number of recent studies have disclosed a critical role for the cell surface Piezo1 channel or, more specifically, Piezo1-mediated Ca^2+^ influx, in the regulation of a multiple of cell functions (e.g., Li et al., 2014; Pathak et al., 2014; Cinar et al., 2015; Hung et al., 2016; Gudipaty et al., 2017; Del Marmol et al., 2018; Friedrich et al., 2019; Mousawi et al., 2019; Solis et al., 2019; Song et al., 2019; Velasco-Estevez et al., 2019; see a recent review by Xiao, 2019). As discussed next, accumulating evidence supports that ATP release and subsequent activation of the P2X and/or P2Y receptors are critical in mediating Piezo1 channel-dependent mechanoregulation.

### Stretch-Induced Piezo1-Dependent Adenosine Triphosphate Release From Urothelial Cells and Regulation of Bladder Function

**Figure 2:**
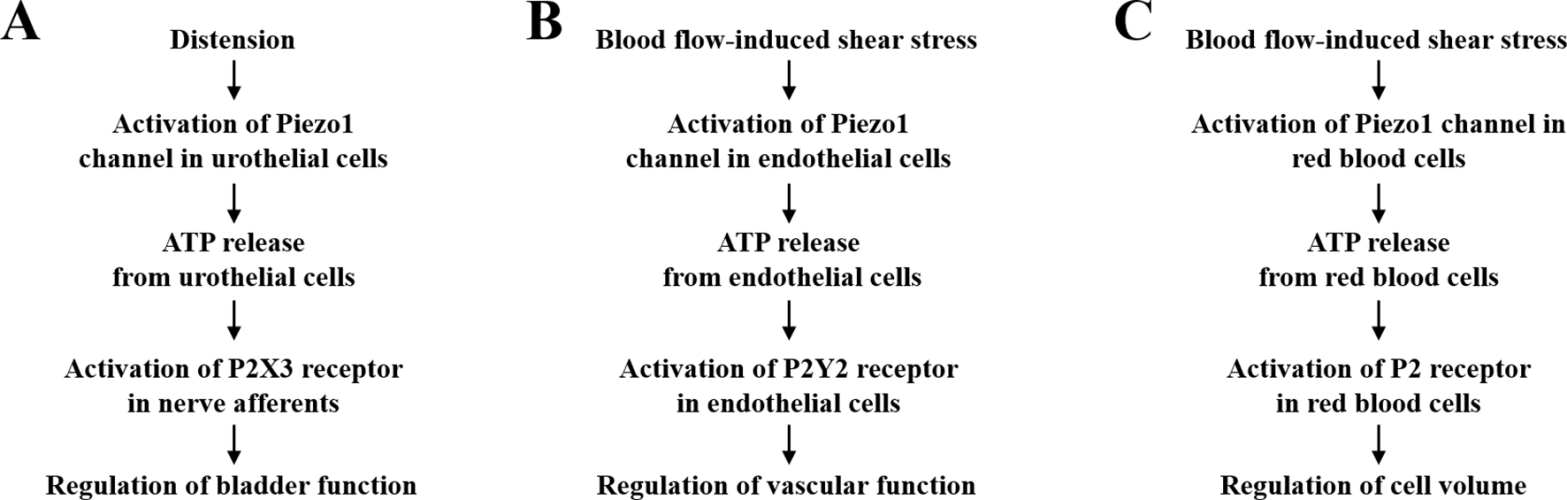
Adenosine triphosphate (ATP) release and activation of P2 receptors in Piezo1 channel-dependent mechanoregulation. **(A)** The Piezo1 channel in urothelial cells senses mechanical forces resulting from bladder distension and induces urothelial cells to release ATP, which in turn acts as a paracrine signal to excite the pelvic nerve afferents by activating the P2X3 receptor. Such signaling mechanism is critical for maintaining the normal bladder function. **(B)** The Piezo1 channel in endothelial cells mediates blood flow-induced release of ATP that serves an autocrine signal acting on the P2Y2 receptor to regulate vascular function and blood pressure. **(C)**. The Piezo1 channel in red blood cells mediates shear stress-induced release of ATP as an autocrine signal to activate yet identified P2 receptor(s) to regulate cell volume. See text for various molecular mechanisms that are known to mediate ATP release.

It is known that as the urinary bladder distends, the urothelial cells become stretched and, as a result, release ATP, which in turn excites the innervating pelvic nerve afferents (Ferguson et al., 1997; Vlaskovska et al., 2001; Beckel et al., 2015). The P2X3 receptor is expressed in the pelvic nerves, and the excitability of the pelvic nerve afferents induced by bladder distension was strongly attenuated in the P2X3-knockout mice, supporting a major role of the P2X3 receptor in transducing ATP release from urothelial cells to excitation of the pelvic nerve afferents (Vlaskovska et al., 2001). Both VNUT-dependent vesicular ATP release *via* exocytosis and ATP efflux through the CALHM1 and pannexin-1 hemi-channels have been shown to mediate ATP release from urothelial cells in response to mechanical forces (Beckel et al., 2015; Nakagomi et al., 2016; Sana-Ur-Rehman et al., 2017). Furthermore, transient receptor potential (TRP) channels, particularly the TRPV4 channel, were suggested to sense mechanical stretch to induce ATP release from urothelial cells (Mochizuki et al., 2009; Merrill et al., 2016). However, compelling evidence indicates that mechanical activation of the TRPV4 channel is indirect, depending on mechanical induction of phospholipase A2-mediated generation of arachidonic acid and/or P450 epoxygenase-mediated generation of 5′,6′-epoxyeicosatrienoic acid from arachidonic acid (Vriens et al., 2004; Berna-Erro et al., 2017). Thus, the molecular mechanism that directly senses mechanical stimuli to trigger ATP release from urothelial cells remained elusive. A recent study has shown expression of the Piezo1 channel in urothelial cells from both human and mouse bladders (Miyamoto et al., 2014). In addition, membrane stretch induced a Ca^2+^ influx-dependent increase in the [Ca^2+^]_i_ in mouse urothelial cells, and such Ca^2+^ response was strongly attenuated by treatment with GsMTx4 or small interference RNA (siRNA)-mediated knockdown of the Piezo1 expression. The same study has further found that stretch stimulated ATP release from mouse urothelial cells. Importantly, stretch-induced ATP release was dependent of extracellular Ca^2+^ and was suppressed by treatment with GsMTx4 or by siRNA-mediated knockdown of the Piezo1 expression. This recent study, taken together with the previous study identifying the P2X3 receptor in coupling urothelial ATP release to pelvic nerve afferent activation (Vlaskovska et al., 2001), supports the notion that the Piezo1 channel in urothelial cells sense the bladder distension and triggers ATP release from urothelial cells and that ATP in turn acts as a paracrine signal excites the pelvic nerve afferents *via* activation of the P2X3 receptor (**Figure 2A**). In other words, the Piezo1 channel in urothelial cells and P2X3 receptor in sensory neurons are important duo players, linked by ATP release from urothelial cells, to maintain the normal bladder function.

### Shear Stress-Induced Piezo1-Dependent Adenosine Triphosphate Release From Endothelial Cells and Regulation of Vascular Function

The vascular endothelium experiences dynamic blood flow-induced shear stress. It is well recognized that the ability of endothelial cells to sense and respond to shear stress is vital for development, function, and disease of the vascular system (Hahn and Schwartz, 2009; Tarbell et al., 2014; Baeyens et al., 2016). ATP release from endothelial cells in response to shear stress has been well documented, and there is compelling evidence to support a critical role of the pannexin-1 hemi-channel in mediating shear stress-induced ATP release (Wang et al., 2015; Wang et al., 2016; Sathanoori et al., 2017). A recent study has shown that ATP released from endothelial cells upon exposure to shear stress serves as a paracrine signal that activates the P2Y2 receptor and downstream signaling pathways, including endothelial nitric oxide synthase to generate nitric oxide (NO), to induce vasodilation (Wang et al., 2015). Consistently, endothelium-specific deletion of the P2Y2 receptor expression in mice led to loss of blood flow-induced vasodilation, resulting in hypertension (Wang et al., 2015). A more recent study from the same group has examined the role of the Piezo1 channel in mediating shear stress-induced ATP release from endothelial cells (Wang et al., 2016). Exposing endothelial cells to shear stress or Yoda1 induced robust Ca^2+^ responses and ATP release, both of which were significantly attenuated by siRNA-mediated knockdown of the Piezo1 expression. ATP release induced by shear stress or Yoda1 was also suppressed by siRNA-mediated reduction in the expression of pannexin-1 or pannexin-2, indicating that shear stress-induced Piezo1-dependent ATP release is at least in part mediated by the pannexin hemi-channels (Wang et al., 2016). Perfusion of the mouse mesenteric arteries or exposure to Yoda1 induced vasodilation, which was impaired by endothelium-specific deletion of the Piezo1 expression. Furthermore, endothelium-specific and conditional knockout of the Piezo1 expression led to elevated blood pressure in mice (Wang et al., 2016), as observed for endothelium-specific and conditional knockout of the P2Y2 receptor (Wang et al., 2015). Collectively, these studies provide compelling evidence to support a vital role of the Piezo1 channel in mediating blood flow-induced release of ATP from endothelial cells as an autocrine signal to regulate the vascular function *via* activating the P2Y2 receptor (**Figure 2B**).

### Shear Stress-Induced Piezo1-Dependent Adenosine Triphosphate Release From Red Blood Cells and Regulation of Cell Volume

Like endothelial cells, red blood cells in circulation are exposed to considerable flow-induced shear stress. Hereditary stomatocytosis and hereditary xerocytosis are rare genetic disorders characterized by red blood cell dehydration. Several gain-of-function mutations in the Piezo1 channel have been shown to be causatively associated these conditions, highlighting a crucial role of the Piezo1 channel in maintaining the normal red blood cell homeostasis (Zarychanski et al., 2012; Albuisson et al., 2013; Bae et al., 2013; Glogowska et al., 2017; Andolfo et al., 2018; Ma et al., 2018). Both human and mouse red blood cells are reported to express the Piezo1 channel on the cell surface. Interestingly, membrane stretch elicited strong Ca^2+^ influx-dependent increase in the [Ca^2+^]_i_ in red blood cells isolated from wild-type mice, but not from mice with conditional knockout of the Piezo1 expression (Cahalan et al., 2015). Similarly, exposure to Yoda1 induced Piezo1-dependent Ca^2+^ entry in mouse red blood cells (Cahalan et al., 2015). Fluid flow-induced shear stress also elicited robust Ca^2+^ influx in human red blood cells, which was significantly suppressed by treatment with GsMTx4, RR or Gd^3+^ (Cinar et al., 2015). Furthermore, genetic deletion of the Piezo1 expression led to red blood cell over-hydration and increased mechanical fragility both *in vitro* and *in vivo*. Conversely, Yoda1-induced activation of the Piezo1 channel caused red blood cell dehydration (Cahalan et al., 2015). These findings demonstrate an indispensable role of the Piezo1 channel in regulating red blood cell function and reveal the Piezo1 channel as a promising target for the development of therapeutics to treat hereditary stomatocytosis and hereditary xerocytosis.

It is long known that red blood cells release ATP in response to mechanical stimuli, such as osmotic stress (Petruzzi et al., 1994) and shear stress (Wan et al., 2008). A previous study showed that ATP release under *in vitro* conditions remained constant in response to shear stress below a certain threshold, but increased significantly above the threshold, which was accompanied with cellular deformation (Wan et al., 2008). A subsequent study provides evidence to suggest that the pannexin-1 hemi-channel is the main pathway mediating ATP release induced by shear stress both above and below the threshold, whereas the CFTR is engaged in deformation-dependent ATP release (Forsyth et al., 2011). A recent study shows that shear stress-induced ATP release was strongly correlated with extracellular Ca^2+^ concentration (Cinar et al., 2015). Shear stress-induced ATP release as well as Ca^2+^ influx in human red blood cells was attenuated by treatment with GsMTx4, RR or Gd^3+^ (Cinar et al., 2015). These results suggest that the Piezo1 channel is important in mediating induction by shear stress of ATP release from red blood cells (**Figure 2C**). Several ATP-sensitive P2 receptors, including P2X1, P2X7, and P2Y1, P2Y11 are expressed in red blood cells, and evidence exists to support that activation of these P2 receptors in red blood cells stimulates a number of signaling pathways that is critical for cell functions, including cell volume regulation (Sluyter, 2015). However, it has not been ascertained which P2 receptor(s) participate(s) in Piezo1-dependent regulation of red blood cell functions.

### Piezo1-Dependent Adenosine Triphosphate Release From Mesenchymal Stem Cells and Regulation of Cell Migration

Mesenchymal stem cells (MSCs), which have promising applications in tissue regeneration and cell-based therapies, are highly mechanosensitive (Engler et al., 2006; Riddle et al., 2007; Shih et al., 2011; Choi et al., 2012; Yang et al., 2012; Yuan et al., 2012; Suhr et al., 2013; Yuan et al., 2013; Chen et al., 2018; Goetzke et al., 2018; Li et al., 2018). It is well recognized that MSCs release ATP in response to mechanical stimulation both *in vitro* and *in vivo* (Riddle et al., 2007; Sun et al., 2013; Weihs et al., 2014). It is also known that several P2X and P2Y receptors are expressed in MSCs and mediate ATP-induced regulation of cell proliferation, migration, and differentiation (Coppi et al., 2007; Riddle et al., 2007; Sun et al., 2013; Peng et al., 2016; Jiang et al., 2017a; Jiang et al., 2017b). A previous study using bone marrow-derived MSCs suggests that fluid flow-induced ATP release *via* the pannexin hemi-channels and subsequent activation of the ATP-sensitive P2Y receptors increased cell proliferation (Riddle et al., 2007). A more recent study shows that shockwave-induced ATP release *via* undefined release mechanisms and subsequent activation of the P2X7 receptor stimulated osteogenic differentiation (Sun et al., 2013). The expression of the Piezo1 channel has been documented in several very recent studies using MSCs from different species and tissues (Gao et al., 2017; Sugimoto et al., 2017; Mousawi et al., 2019). Our recent study shows that Yoda1-induced activation of the Piezo1 channel in human dental pulp MSC promoted migration, which was suppressed by siRNA-mediated knockdown of the Piezo1 expression (Mousawi et al., 2019). More importantly, Yoda1-induced activation of the Piezo1 channel stimulated ATP release from human dental pulp MSCs (Mousawi et al., 2019). Yoda1-induced Piezo1-dependent increase in cell migration was inhibited by treatment with apyrase, a scavenger of extracellular ATP, and also with PPADS, a P2 receptor generic antagonist. Taken together, these results support the notion that activation of the Piezo1 channel enhances MSC migration *via* inducing release of ATP as an autocrine signal that activates the P2 receptors. Our previous study has identified P2X7, P2Y1, and P2Y11 as the major P2 receptors that participate in mediating ATP-induced stimulation of human dental pulp MSC migration (Peng et al., 2016). It is highly interesting to examine the role of ATP release and the P2 receptors in Piezo1-dependent mechanoregulation of MSC functions such as differentiation and migration.

### Adenosine Triphosphate Release and P2 Receptor as a Common Signaling Mechanism in Piezo1 Channel-Dependent Mechanoregulation?

As mentioned above, recent studies demonstrate expression of the Piezo1 channel in many different types of mechanosensitive cells with an important role in the mechanoregulation of attendant cell functions. The majority, if not all, of these cells, are known to express the P2X/P2Y receptors that are important in mediating ATP-induced regulation of their functions. This raises the perspective that ATP release integrates the Piezo1 channel and P2 receptor as a more common signaling mechanism in the mechanoregulation of cell functions.

The expression of the Piezo1 channel is required for alignment of endothelial cells in response to shear stress (Li et al., 2014; Ranade et al., 2014). Similarly, the P2Y2 receptor in endothelial cells plays an important role in mediating shear stress-induced cell alignment (Sathanoori et al., 2017). It is interesting to investigate whether shear stress-induced ATP release couples the Piezo1 and the P2Y2 receptor in the regulation of vascular development. Another recent study shows that shear stress induces ATP release from red blood cells, on one hand, and an increase in the [Ca^2+^]_i_ and NO generation in endothelial cells and formation of inter-endothelial junctions, on the other. These shear stress-induced responses or effects both in red blood cells and endothelial cells were prevented by pharmacological inhibition and genetic depletion of the pannexin-1 channel on red blood cells (Xu et al., 2017). It is unknown whether shear stress-induced Piezo1-dependent ATP release from red blood cells acts as a paracrine signal to induce Ca^2+^ signaling in endothelial cells *via* activating the P2X/P2Y receptors.

As discussed above, ATP release coupling of the Piezo1 channel in urothelial cells and the P2X3 receptor in the pelvic nerve afferents is important in maintaining the normal bladder function. Such a signaling mechanism may also play an important role in mediating dentinal pain. It is known that dentinal fluid-induced odontoblast deformation can evoke dentinal pain. A recent electrophysiological study shows that pressure-induced odontoblast deformation elicited inward currents that caused membrane depolarization and induced action potentials in co-cultured isolectin IB4-negative medium-sized trigeminal ganglion neurons (Sato et al., 2018). Furthermore, such inward currents were significantly attenuated by treatment with NF110, a P2X3 receptor antagonist, or with GsMTx4 as well as with a cocktail of TRP channel inhibitors (Sato et al., 2018). It is thus hypothesized that Piezo1/TRP-dependent ATP release from odontoblasts in response to mechanical stimulation excites myelinated Aδ neurons *via* activating the P2X3 receptor, thereby forming a signaling mechanism generating dentinal pain.

Cancer cells in the metastasis process encounter mechanical forces such as compression from the surrounding extracellular matrix and cells in the primary site, invasion into neighboring tissues, intravasation and extravasation through endothelial cells, micro-metastasis at target tissues or organs. They also experience blood flow-induced shear stress during circulation in the blood stream. It is conceivable that mechanical forces influence cancer cell migration, invasiveness, and metastasis. Consistently, several recent studies provide increasing evidence to show that activation of the Piezo1 channel stimulates cell proliferation in gastric cancer cells (Zhang et al., 2018), and enhances cell migration in gastric cancer cells (Yang et al., 2014; Zhang et al., 2018) and malignant MCF-7 breast cancer cells (Li et al., 2015) but reduces non-small cell lung cancer progression and cell migration (Huang et al., 2019). Compelling evidence already exists to support that extracellular ATP can regulate cancer cell migration, invasiveness, and metastasis *via* activating the P2X7, P2Y2 or P2Y11 receptors (Jelassi et al., 2011; Jelassi et al., 2013; Schumacher et al., 2013; Chadet et al., 2014; Roger et al., 2015; Khalid et al., 2017). Particularly, it was shown that ATP released from platelets bound to the circulating cancer cells and consequently activates the P2Y2 receptor on endothelial cells to promote formation of inter-endothelial junctions for cancer cell migration (Schumacher et al., 2013). As discussed above, shear stress can induce ATP release from red blood cells. It is attractive to speculate that shear stress-induced ATP release from red blood cells acts as a paracrine signal to induce formation of inter-endothelial junctions *via* activating the P2X/P2Y receptors in endothelial cells and thereby facilities intravasation and extravasation of cancer cells.

## Concluding Remarks

It is evident from the discussion above that accumulating evidence supports an important role of ATP release as an autocrine and/or paracrine signal and subsequent activation of the P2 receptors in Piezo1 channel-dependent mechanoregulation of cell functions and associated physiological processes (**Figure 2**). As illustrated by hereditary stomatocytosis and hereditary xerocytosis, alterations in such signaling mechanisms resulting from mutations in the Piezo1 channel in red blood cells can lead to cell dysfunction and severe human diseased conditions. Studies so far support the Piezo1 channel as an intrinsic mechanosensor to trigger ATP release in response to mechanical stimulation. However, it remains unknown how activation of the Piezo1 channel regulates the ATP release mechanisms. Finally, more investigations are required to determine whether the Piezo1 channel and P2 receptors coupled by ATP release form a common signaling mechanism in transducing mechanical force information to regulation of cell functions.

## Author Contributions

All authors contributed to the development of the concept. L-HJ wrote the manuscript. All the authors commented and approved the manuscript.

## Conflict of Interest

The authors declare that the research was conducted in the absence of any commercial or financial relationships that could be construed as a potential conflict of interest.
